# Artificial Intelligence for In-Flight Detection of Space-Related Ocular Trauma: Bridging Diagnostic Gaps in Microgravity

**DOI:** 10.3390/vision10040059

**Published:** 2026-08-26

**Authors:** Jason Zheng, Jainam Shah, Sachin Pathuri, Joshua Ong, Andrew G. Lee

**Affiliations:** 1 California University of Science and Medicine, Colton, CA 92324, USA; jason.zheng@md.cusm.edu; 2 Albert Einstein College of Medicine, Bronx, NY 10461, USA; jainam.shah@einsteinmed.edu; 3 Creighton University School of Medicine, Phoenix Regional Campus, Phoenix, AZ 85012, USA; sachinpathuri@creighton.edu; 4 Department of Ophthalmology, Harvard Medical School, Massachusetts Eye and Ear, Boston, MA 02114, USA; joshuaong.md@gmail.com; 5 Department of Ophthalmology, Blanton Eye Institute, Houston Methodist Hospital, Houston, TX 77030, USA; 6 Department of Ophthalmology, Baylor College of Medicine and the Center for Space Medicine, Houston, TX 77030, USA; 7 The Houston Methodist Research Institute, Houston Methodist Hospital, Houston, TX 77030, USA; 8 Departments of Ophthalmology, Neurology, and Neurosurgery, Weill Cornell Medicine, New York, NY 10021, USA; 9 Department of Ophthalmology, University of Texas Medical Branch, Galveston, TX 77555, USA; 10 University of Texas MD Anderson Cancer Center, Houston, TX 77030, USA; 11 Texas A&M College of Medicine, Bryan, TX 77807, USA; 12 Department of Ophthalmology, The University of Iowa Hospitals and Clinics, Iowa City, IA 52242, USA; 13 Department of Ophthalmology, University of Buffalo, Buffalo, NY 14209, USA

**Keywords:** space medicine, spaceflight, artificial intelligence, ocular trauma, ocular management

## Abstract

Ocular trauma represents a threat to crew safety and mission performance in space. Microgravity, confined environments, and exposure to particulate matter, chemicals, and mechanical hazards place astronauts at risk for corneal abrasions, open-globe injuries, chemical burns, lens dislocation, retinal detachment, orbital fractures, and barotrauma. Diagnostic capabilities during spaceflight remain limited by resources, lack of specialist expertise, and communication delays with Earth. Artificial intelligence, particularly convolutional neural networks and multimodal models, may help address these gaps through image interpretation, risk stratification, and longitudinal monitoring. Convolutional neural networks can extract hierarchical features from imaging data to identify subtle structural abnormalities, while multimodal models integrate imaging with clinical and environmental parameters to generate more comprehensive assessments. Terrestrial ophthalmology studies demonstrate the potential of these approaches across optical coherence tomography, ultrasound, fundus photography, and anterior-segment imaging. This review examines how these capabilities can be matched to ocular injuries during spaceflight, compares the suitability of different approaches across injury types, and identifies pathways toward autonomous care. Particular emphasis is given to spaceflight-related imaging and physiologic changes, constrained onboard hardware, and integration into workflows that support non-expert crew members. Collectively, these applications may expand diagnostic capabilities and enable earlier, more informed management during long-duration missions.

## 1. Introduction

### 1.1. Ocular Health in Space

As space agencies such as the National Aeronautics and Space Administration (NASA) pursue longer distance and extended duration flights, maintaining crew health will become an increasingly important consideration [1,2]. The microgravity spaceflight environment is associated with numerous physiologic effects, including bone demineralization [1,3,4], muscle loss [1,5], cardiac atrophy [6,7], and visual impairments [8,9], while debris, chemicals, equipment, and other onboard hazards create an additional risk of ocular trauma [10]. NASA data from the Life Sciences Data Archive (LSDA) and the Lifetime Surveillance of Astronaut Health (LSAH) revealed nearly 250 cases of ocular complaints across both International Space Station (ISS) and Space Shuttle (SHT) missions, including substantial ocular trauma, most commonly ocular foreign bodies [11]. Although none of these cases resulted in mission termination, ocular trauma, even when seemingly minor, may compromise mission performance, crew safety, and long-term visual health if not promptly and accurately assessed [11]. These reports underscore the need for reliable evaluation and management of ocular injury during spaceflight.

**Figure 1:**
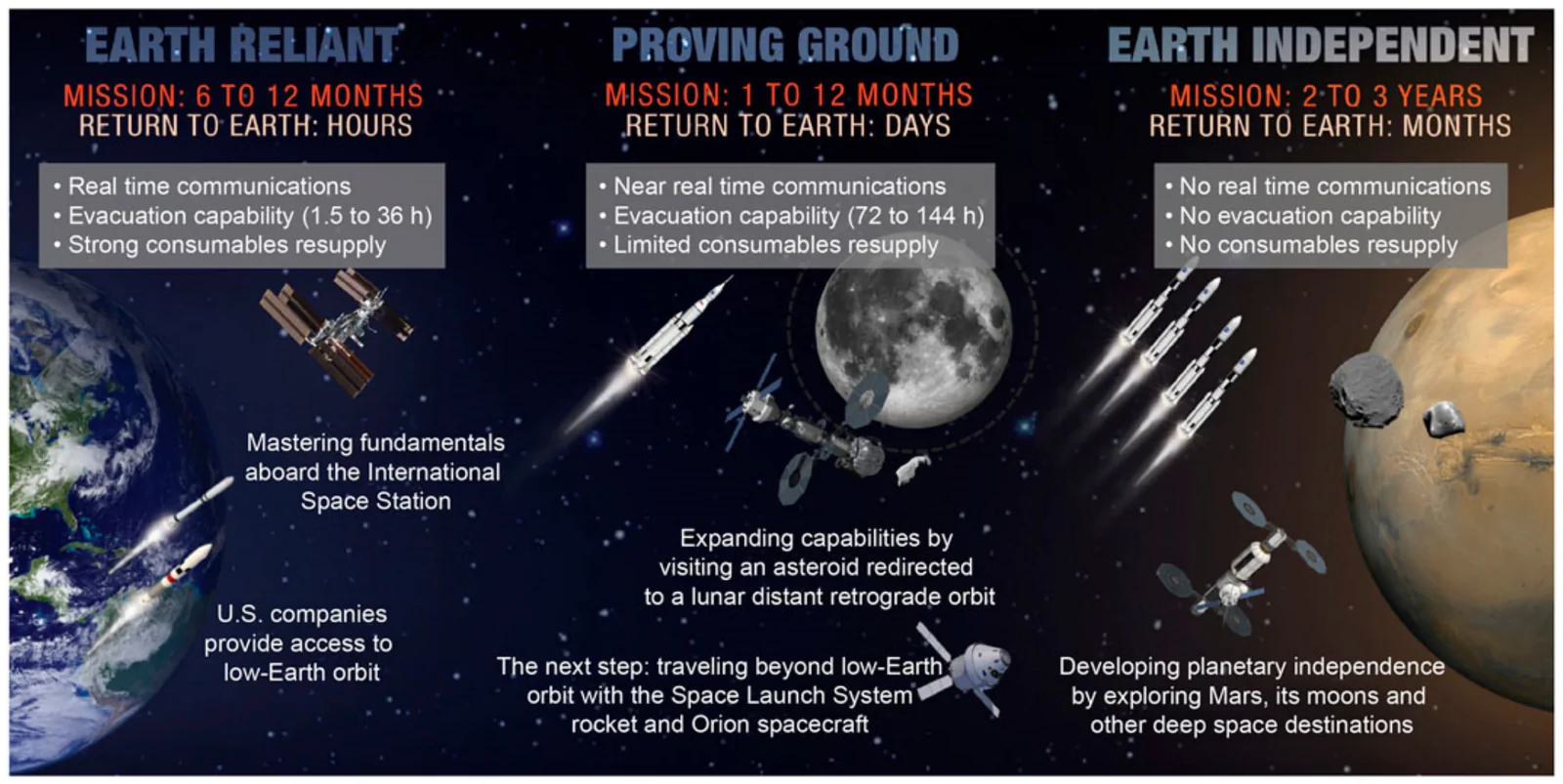
Graphical illustration of the communication delays and evacuation challenges associated with increasingly longer and distant space missions. Reprinted with permission from ref [16].

On Earth, clinicians have access to a broad range of imaging modalities to diagnose and monitor ocular injuries, whereas diagnostic capabilities aboard spacecraft are more limited. Available onboard tools include tonometers, ophthalmoscopes, fluorescein strips, blue-light filters, optical coherence tomography (OCT), ocular ultrasonography, and OCT angiography [12,13]. Furthermore, as mission distance increases, communication delays may increasingly limit real-time consultation and telemedical guidance between the crew and ground-based medical support teams [14,15]. As timely Earth-based guidance and medical evacuation become less feasible, crews may need to independently assess ocular injuries and guide initial management using available onboard tools (Figure 1) [16].

Artificial intelligence (AI) may help address this gap by supporting interpretation of ophthalmic data and assisting with the detection, triage, and monitoring of ocular injuries when real-time specialist input is unavailable. However, no AI-based ocular diagnostic system has been validated during spaceflight, and current evidence is derived from terrestrial studies. Accordingly, this review does not assume that terrestrial performance translates directly to spaceflight. We instead evaluate AI approaches by matching demonstrated capabilities to specific diagnostic needs across ocular injuries, comparing the applicability and limitations of the available evidence, and assessing their translational readiness across three domains: adaptation to spaceflight-related differences in imaging and physiology, feasibility on constrained onboard hardware, and integration into an autonomous clinical workflow. We first review convolutional neural networks and multimodal approaches relevant to ophthalmic imaging, then apply this framework across major categories of ocular trauma and conclude by examining the data, technical, ethical, and operational barriers that must be addressed before potential spaceflight deployment.

### 1.2. Methodology

This is a narrative review; no formal systematic review protocol (e.g., PRISMA) was followed, given the heterogeneity of the clinical, engineering, and space-medicine literature synthesized here. Relevant publications were identified through searches of PubMed and Google Scholar, conducted from April 2026 through June 2026 using combinations of the terms “spaceflight,” “astronaut,” or “microgravity” with “ocular trauma,” “ocular injury,” “corneal abrasion,” “open-globe injury,” “retinal detachment,” “orbital fracture,” or “barotrauma,” and with “artificial intelligence,” “deep learning,” “convolutional neural network,” or “multimodal model.” Reference lists of retrieved articles were hand-searched for additional relevant studies. We prioritized peer-reviewed publications reporting quantitative diagnostic performance (e.g., accuracy, sensitivity, specificity, area under the receiver operating characteristic curve [AUROC]) for AI models applied to ophthalmic imaging modalities available or plausible in a spaceflight setting (fundus photography, OCT, ultrasound, and slit-lamp imaging). Non-English-language publications were excluded.

## 2. Artificial Intelligence Applications in Ophthalmic Imaging

AI systems can analyze large volumes of medical data, detect subtle anatomic abnormalities that may indicate early ocular pathology, and enhance degraded or low-resolution images [17]. These capabilities may be valuable when only compact, lower-fidelity imaging devices are available during missions. Among AI systems, convolutional neural networks (CNNs) and multimodal models are especially relevant to ophthalmology. CNNs are deep learning models that analyze images by identifying spatial patterns within visual data. Through layered filters, they first detect simple features such as edges and contrast and then progressively combine these signals to recognize more complex structures, including retinal layers, blood vessels, and pathological lesions. Multimodal models extend this approach by integrating multiple types of data, including different imaging modalities and clinical variables such as visual acuity or intraocular pressure (IOP), allowing the model to learn relationships between complementary data sources and produce a more comprehensive representation of disease. Given the breadth of AI modalities, this review will focus specifically on multimodal models and CNNs, as they are the most widely used and relevant approaches to ophthalmology.

CNNs have been widely applied to ophthalmic imaging modalities such as fundus photography and OCT. For example, a CNN model trained on retinal photographs for diabetic retinopathy detection achieved an AUROC of 0.991 [18]. Multimodal deep learning systems have also demonstrated strong predictive performance, with a recent study reporting an AUROC of 0.94 in a model designed for glaucoma detection [19]. Several multi-task and multi-scale feature-fusion frameworks developed outside ophthalmology have illustrated design strategies that may inform future ocular-trauma models. For example, YOLO-TCS addressed the difficulty of detecting small, distant traffic signs within visually cluttered environments by using multi-scale feature fusion and an attention mechanism to prioritize relevant image regions, improving detection of these subtle targets [20]. Separately, a “multi-task” model developed for criminal investigation used a single thermal handprint image to both identify an individual and estimate the time since surface contact [21,22]. Although these models were not developed for ophthalmic or space-medicine applications, their design principles, including handling multiple tasks within one model, integrating coarse and fine image detail, and prioritizing relevant input features, offer precedent for future ocular-trauma models that integrate ocular imaging with other spaceflight data streams. Their relevance may lie in transferable architectural strategies rather than evidence of direct applicability to ocular care in space.

Spaceflight-associated changes in ocular anatomy and physiology may alter the imaging features used by AI models to distinguish normal from abnormal findings. CNNs can identify structural patterns within imaging data, while multimodal systems incorporate additional physiological or environmental inputs, enabling detection of subtle deviations from baseline ocular anatomy and physiology. Their relative value may therefore depend on the diagnostic task, as CNNs may be better suited to injuries characterized by recognizable structural abnormalities. Multimodal models, on the other hand, may offer greater value when assessment requires integrating imaging with physiologic, clinical, or environmental information.

Importantly, no CNN- or multimodal AI-based diagnostic system has been developed, tested, or validated specifically within an actual spaceflight environment. Every performance metric discussed in this review is derived from terrestrial studies and should not be interpreted as expected in-flight performance. Evidentiary maturity also varies substantially by injury type, as some ocular injuries are supported by models with relatively well-characterized terrestrial validation cohorts, whereas others have no dedicated ocular AI model. Model speed and computational footprint, factors directly relevant to onboard deployment, are also rarely reported. These differences limit direct numerical comparison across studies but do not preclude comparison of their suitability for specific ocular injuries. Accordingly, the sections below evaluate each approach according to the diagnostic task addressed, the directness and limitations of the supporting evidence, and its requirements for potential onboard use.

We evaluate the translation of terrestrial AI toward spaceflight use across three domains: data and physiological domain-shift adaptation, hardware and edge-computation feasibility, and integration into an autonomous clinical workflow. The relative importance of each domain depends on the maturity and intended function of the application. The first, data and physiological domain-shift adaptation, addresses whether statistical patterns learned from terrestrial images remain reliable when the input distribution changes because of degraded or non-standard image acquisition, including handheld sensors, motion artifact, and inconsistent illumination. Spaceflight-associated changes in ocular anatomy and physiology may create an additional source of domain shift. Closing this gap may require retraining on spaceflight-representative data or, where such data are scarce, deliberately simulating expected shifts within terrestrial datasets. The second domain, hardware and edge-computation feasibility, addresses whether a model validated using terrestrial clinical-grade computing can operate within the size, weight, and power constraints of spacecraft hardware. This may require compression through quantization or pruning, followed by testing to determine whether performance is preserved on representative onboard hardware. Terrestrial diagnostic performance and operational feasibility represent separate requirements for translation. The third domain, autonomous workflow, addresses how model output becomes an actionable clinical decision when the person interpreting it is not a specialist and real-time ophthalmic consultation may be unavailable. This may require graded outputs, identification of uncertainty, and decision thresholds tied to observation, treatment, or escalation/emergency protocols. Clinical utility ultimately depends not only on identifying an abnormality, but also on providing information that can safely support the subsequent decision required of the crew. These domains provide the framework used below to distinguish demonstrated terrestrial capabilities from potential spaceflight applications and identify the remaining requirements for translation.

## 3. Ocular Trauma

### 3.1. Lunar Dust and Corneal Abrasions

**Figure 2:**
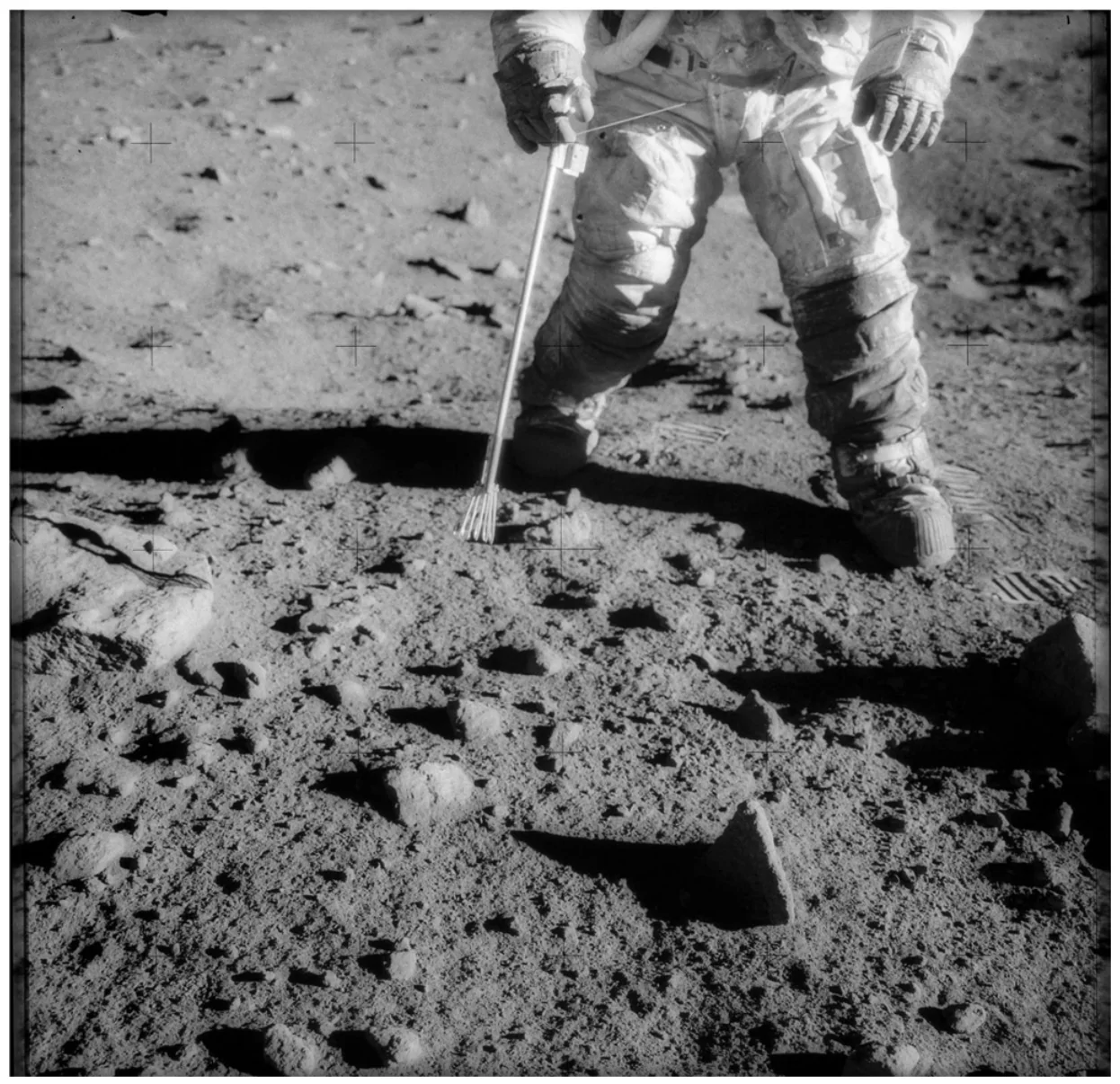
The moon’s surface is covered by layers of rocks, dust, and debris known as regolith. As the astronaut walked on the moon, lunar dust started collecting on the spacesuit. Reprinted with permission from ref [23].

Exposure to lunar dust is especially important to address because the most common cause of eye injury in space is corneal abrasion secondary to foreign bodies. Astronauts can encounter lunar dust through extravehicular activities (EVAs), including spacewalks and spacecraft repairs (Figure 2) [23]. During EVAs, degradation of the suit may enable entrapment of space debris within the suit while lunar dust adhering to spacesuits and boots can be carried into the spacecraft. In microgravity, these particles become suspended and freely circulate with cabin airflow, increasing the likelihood of ocular exposure and injury. Microgravity may also alter normal tear film distribution across the cornea, promoting dryness and increasing susceptibility to abrasions [24,25]. Although short-term studies of lunar dust have not demonstrated significant ocular irritation, its longer-term effects and those of other celestial particulates remain unknown and should be further analyzed [23].

Small corneal abrasions or conjunctival/corneal foreign bodies are commonly diagnosed using slit-lamp biomicroscopy in combination with fluorescein staining. On the ISS, where slit lamp microscopes are currently not available, corneal abrasions are diagnosed via ophthalmoscopes, fluorescein strips, blue light filter, and anterior segment optical coherence tomography (AS-OCT) [12,26]. Advances in automated analysis of corneal fluorescein staining and anterior segment imaging may streamline this diagnostic process in spaceflight environments. CNNs have demonstrated superior performance to junior clinicians in detecting epithelial defects from fluorescein-enhanced slit-lamp images, accurately quantifying both the area and severity of corneal damage, with an accuracy of 91.2% [27]. Similar segmentation algorithms could be adapted for spaceflight to autonomously identify epithelial disruptions characteristic of corneal abrasions from the available diagnostic tools. CNN image diagnostic models, such as CorneAI, have also significantly improved clinicians’ overall diagnostic accuracy by 9.6% for anterior segment diseases, even when interpreting lower-quality images captured by smartphone [28]. Although the model has not yet been validated for detecting corneal abrasions specifically, its adaptability suggests potential for spaceflight applications. Translation would also require matching the model to a flight-compatible imaging platform. The fluorescein-based CNN was developed using slit-lamp images, whereas CorneAI demonstrates analysis of images acquired using a more portable smartphone platform. A spaceflight system would need to preserve abrasion-detection performance using an onboard imaging device and demonstrate that the model can execute locally within its computational constraints. The two models address complementary aspects of the diagnostic problem: the fluorescein-based CNN provides more direct evidence for detecting and characterizing epithelial defects, while CorneAI provides less injury-specific evidence but demonstrates feasibility with lower-quality, portable imaging. Neither has yet combined abrasion-specific detection with an imaging platform representative of spaceflight use. Additionally, with the capabilities of modern sensors, large-scale data such as orbital debris trends and ultraviolet radiation data may be utilized to train CNNs to forecast safer windows for EVAs and minimize exposure to ocular hazards [29]. This preventive application remains more conceptual than image-based abrasion detection and would require validation linking environmental inputs to clinically meaningful ocular risk.

For image-based abrasion detection, domain shift represents a more immediate translational barrier. The smartphone-image model described above has only been evaluated using clear, well-lit photographs. It has not been tested on an image where dust is adherent to the cornea or illumination is uneven, either of which may be mistaken by the model for a genuine corneal opacity or foreign body. A plausible approach would be to deliberately alter existing training images before retraining, including overlaying simulated dust particles, reproducing cabin lighting, and adding the graininess a small flight-qualified camera may produce. The model would then be retrained on these altered images, and its accuracy compared with performance on the original, clear images. Because dust exposure happens specifically around spacewalks, the model could also incorporate recent activity, such as time since returning from an EVA, so that it weighs a corneal finding differently depending on whether dust exposure was likely. Testing an abrasion-specific model with flight-compatible imaging under these simulated conditions would provide a more direct assessment of translational readiness than extrapolating from terrestrial accuracy alone.

**Figure 3:**
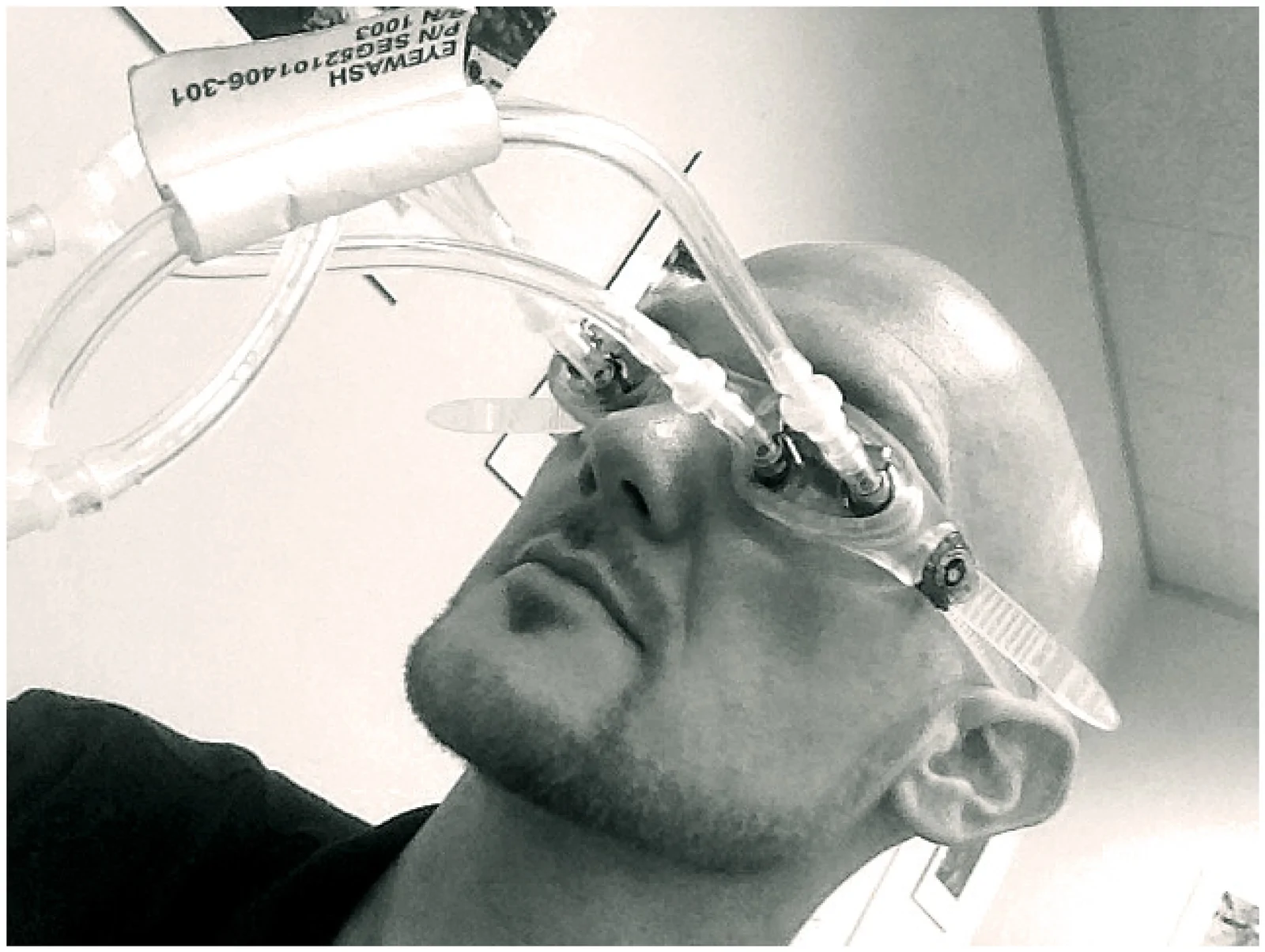
Photograph of an astronaut using NASA’s space eye wash. Goggles are connected to an eyewash saline solution that is pumped around the eyes and then away. Reprinted with courtesy of NASA.

Operationally, these algorithms could be embedded directly into handheld anterior-segment imaging systems carried aboard future spacecraft. Rather than transmitting every image to Earth, astronauts could receive immediate estimates of injury severity together with management recommendations and guidance regarding repeat imaging, observation, or escalation of care. Once identified, crew members should irrigate their eyes with the space eye wash (Figure 3) to remove foreign bodies. More persistent and larger-sized foreign bodies should be removed with gentle sweeping of a cotton swab after applying topical anesthetics (proparacaine hydrochloride 0.5% or tetracaine hydrochloride 0.5%). Topical antibiotics such as erythromycin 0.5% should be applied after foreign body removal; contact lens users should be provided antipseudomonal coverage with moxifloxacin 0.5% drops. Cyclopentolate 1%, topical nonsteroidal anti-inflammatory drugs (NSAIDs), and short-acting topical cycloplegic drops may be used to control ocular pain and discomfort [30]. Within this workflow, AI could bridge the diagnostic and triage gap by helping characterize injury severity and determine when established management protocols should be initiated or escalated.

### 3.2. Globe Penetration

While superficial corneal injuries are expected to be the most common ocular trauma encountered during spaceflight, deeper penetrating injuries may present substantially greater diagnostic and management challenges. Open-globe injuries are full-thickness disruptions of the eye caused by trauma and encompass injuries from both blunt and sharp forces, including penetrating and perforating injuries, as well as the entry of intraocular foreign bodies (IOFB) [31,32]. Although no documented cases of open-globe injuries have been reported in spaceflight literature to date, several plausible scenarios exist in which astronauts could sustain severe ocular trauma. For instance, high-velocity particles or larger fragments dislodged from cargo during operations may potentially penetrate the globe or cause significant ocular damage. This concern is reinforced by the 70 reported incidents of ocular foreign bodies during space missions, underscoring the realistic risk of such injuries in the space environment [24].

Diagnosis and management of open-globe injuries would pose substantial challenges aboard spacecraft. While crew members may be able to perform basic penlight examinations to gather clues for ocular injury and use ultrasound to detect IOFBs [24], these methods have important limitations. Ultrasound identifies IOFBs in only about 52% of cases and carries the risk of expelling intraocular contents if excessive pressure is applied [33]. Smaller wounds may require slit-lamp examination with fluorescein staining to confirm full-thickness corneal lacerations or detect anterior chamber foreign bodies; however, slit-lamp examination is unavailable during spaceflight [24]. Delays in repair may worsen visual outcomes, highlighting the critical need to accurately identify these ocular injuries in the resource-limited environment that crew members face [31]. Open-globe injury therefore presents a different diagnostic problem from corneal abrasion because the available onboard examination may be insufficient to exclude a subtle but vision-threatening injury, while the consequences of a false-negative assessment are substantially greater.

Multimodal AI systems offer a potential solution to these limitations. Tools such as EE-Explorer can combine ocular surface images captured via smartphone with clinical data to detect these vision-threatening injuries with near-expert performance (AUROC of 0.982), paving the way to facilitate diagnosis even in remote settings [34]. Its combination of portable ocular imaging and clinical data is conceptually compatible with an onboard workflow, although autonomous use would also require local processing of these inputs on flight-compatible hardware without reliance on continuous Earth-based computation. Beyond detection, multimodal AI models that integrate ocular imaging with structured clinical variables have demonstrated the ability to predict future visual outcomes [35]. Perhaps incorporating features such as IOFB presence, wound location, retinal detachment, and presenting visual acuity could offer multimodal models a means to automate risk stratification and triage in ocular trauma. AI may also address the risk of false-negative assessments, which can occur when subtle or incomplete clinical signs such as preserved globe contour, minimal external wounds, or relatively maintained visual acuity mislead non-specialist examiners. By analyzing patterns across multiple discordant signals, including asymmetric anterior chamber depth and disproportionate visual function changes [36], CNN models could be constructed to flag probable globe penetration even in the absence of overt indicators. This is backed by similar pattern-recognition approaches used in terrestrial trauma imaging, which have helped reduce missed diagnoses [37]. Compared with a purely image-based CNN, a multimodal approach may be particularly suited to suspected globe penetration because no single onboard finding reliably excludes the injury and assessment depends on combining imaging, examination findings, visual function, and injury characteristics. The current evidence is strongest for terrestrial detection and outcome prediction, however, while autonomous identification of subtle or atypical spaceflight-related penetrating injuries remains unvalidated.

Domain shift is an important consideration, given the mechanism and appearance of potential spaceflight injuries. EE-Explorer and similar models were trained on emergency-room patients, where injuries commonly result from assaults, car crashes, or accidents at home, which may produce wounds different from injuries caused by spaceborne particles or onboard objects. One practical test would be to utilize images that do not resemble typical emergency room ophthalmic trauma cases and compare performance in this subgroup with overall performance. Because the clinical cost of error is asymmetric, validation should also emphasize sensitivity and false-negative rates rather than overall accuracy alone. A graded risk output could flag borderline cases more readily, even at the cost of additional false-positive assessments. For an injury in which delayed repair worsens visual outcome [31], accepting more false alarms may be preferable to falsely reassuring the crew that globe penetration is absent. This makes autonomous workflow and uncertainty handling at least as important as diagnostic discrimination for this application.

During future lunar and Martian missions, where communication delays may exceed twenty minutes each way, these systems could function as autonomous triage tools that prioritize injuries requiring immediate stabilization versus continued observation until Earth-based consultation becomes available. Their greatest value may lie in facilitating independent medical decision-making rather than simply improving diagnostic accuracy. Following suspected globe penetration, the offending object should not be manipulated. Both eyes should be covered with an eye pad to minimize ocular movement and reduce the risk of further extrusion of intraocular contents. The injured crewmember should be assisted by fellow crew members, and mission control should be contacted immediately to coordinate further management and potential evacuation. Initial management includes antimicrobial prophylaxis, cycloplegic agents, analgesia, and lubrication, with bandage contact lenses or collagen corneal shields offering additional support for epithelial healing and drug delivery. For small corneal perforations less than 3 mm, cyanoacrylate glue may provide temporary globe stabilization until definitive surgical intervention is possible [30]. Within this pathway, AI would primarily support recognition and risk stratification before definitive treatment, helping distinguish injuries that may be observed from those requiring immediate protective measures and escalation.

### 3.3. Chemical Injuries

Beyond mechanical trauma, astronauts must also contend with chemically mediated ocular injury arising from the spacecraft environment. Toxin exposure has been a concern since the beginning of human space exploration. Astronauts may be exposed to corrosive and irritant compounds capable of inducing ocular surface injury, including potassium hydroxide from batteries, chromium trioxide and sulfuric acid from urine pretreatment solutions, lithium hydroxide dust from carbon dioxide scrubbers, and formaldehyde released through material off-gassing [38]. Potassium hydroxide and lithium hydroxide are both alkalis that penetrate deeply into ocular tissues and saponify cell membrane lipids, initiating liquefactive necrosis and release of proteolytic enzymes that further propagate tissue destruction. Consequently, this leads to corneal epithelial loss, stromal opacification, and potentially permanent vision loss [39,40,41]. In contrast, acids cause ocular injury primarily through coagulative necrosis, with coagulated proteins limiting deep ocular penetration [40,42]. However, acid exposures may potentially be as severe as those of alkalis [43]. Environmental factors may also contribute to ocular discomfort. In the absence of convection during spaceflight, carbon dioxide accumulates near the face, causing transient corneal and conjunctival irritation. While not vision-threatening, such discomfort may impair concentration and visual performance during critical operations [38]. Collectively, these exposures underscore the importance of recognizing and mitigating chemical ocular injuries during long-duration missions.

**Figure 4:**
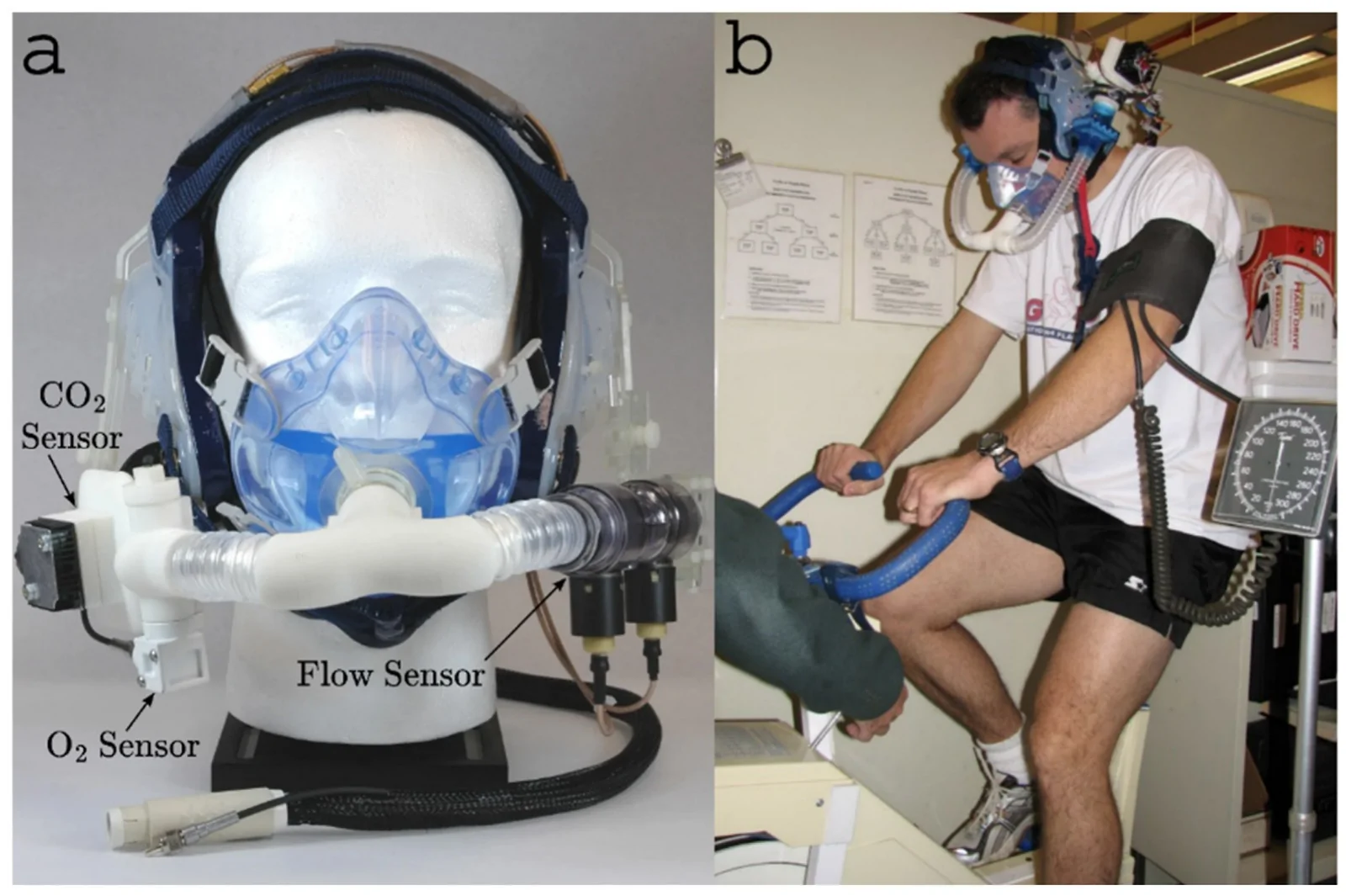
An example of one of NASA’s wearable breath analysis systems used to measure astronauts’ O_2_ inhalation rate, CO_2_ exhalation rate, and total exhalation flow. Its limited breath marker analysis, however, inherently limits the health information that could be provided to astronauts. (**a**) The wearable breath analysis system on a mannequin with key parts labeled. (**b**) The wearable breath analysis system worn by a volunteer. Reprinted with permission from ref. [44].

While solid and liquid chemical hazards can often be detected visually, gaseous compounds present a unique challenge because many are colorless and odorless, making them difficult to detect without dedicated sensors. Yet, they can accumulate in the cabin atmosphere and pose significant risks to crew members. The increasing complexity of space vehicles and the broad range of onboard experiments have made controlling cabin air pollution a significant operational and biomedical challenge. Previously, spaceflight systems were largely limited to O_2_, CO_2_, and flow sensing (Figure 4), and thus could not quantify volatile organic compounds (VOCs) or other biomarkers that convey physiological or pathological information [44]. The air quality monitoring system, Analyzing Interferometer for Ambient Air 2, launched in 2021, represents a major step forward; it can identify and quantify more than 40 gaseous compounds on the ISS and lays the groundwork for integration with automated breath-analysis and wearable chemical sensing systems. For chemical ocular injury, this creates a fundamentally different AI problem from the image-based injuries because environmental sensing may potentially identify a hazardous exposure before ocular damage becomes clinically apparent, while ocular imaging would characterize injury after exposure.

Technologies such as exhaled breath condensate and VOC samplers capture aerosols and gases that mirror blood-borne compounds at lower concentrations, enabling noninvasive physiologic monitoring. In terrestrial studies, these breath-analysis systems have detected conditions such as diabetes and distinguished disease states, including differentiating patients with asthma from healthy controls and identifying COVID-19 infection based on characteristic chemical signatures in breath or skin-emitted VOCs [45]. These noninvasive approaches could provide a foundation for longitudinal astronaut health surveillance by continuously collecting physiologic and environmental data throughout missions. Variables such as temperature, oxygenation, radiation exposure, air quality metrics, and visual or imaging findings could potentially be integrated into multimodal AI models to identify patterns preceding medical complications. Repeated observations could also allow retrospective identification of environmental or operational factors associated with adverse effects and refinement of predictive algorithms for subsequent missions. Over time, such models could provide real-time risk estimates and personalized recommendations, including when crew members should limit activity, disengage from hazardous environments, or modify mission tasks to reduce the likelihood of injury or illness. This may enhance immediate crew safety and generate an evolving evidence base to guide safer exploration on future long-duration voyages. Ultimately, future spacecraft may incorporate continuously learning environmental surveillance systems that integrate cabin chemistry, wearable physiologic sensors, and ocular imaging into a unified AI platform capable of identifying hazardous exposures before clinically significant ocular injury develops. This proposed progression remains substantially more conceptual than the image-based applications discussed above. Current terrestrial evidence demonstrates that VOC-based systems can distinguish certain systemic disease states, but does not establish that chemical ocular injury produces a detectable breath signature or that these inputs can predict ocular toxicity. Establishing that biological link is therefore necessary before such an integrated predictive system can be developed.

A more fundamental limitation is that terrestrial studies have not established whether chemical ocular injury produces a detectable breath signature. Before predictive models can be developed, it must first be determined whether ocular exposure to an alkali, acid, or other relevant compound produces a reproducible signal detectable by available sensors. One approach would be to study populations with comparable occupational chemical exposures and compare their breath composition with that of unexposed controls. If a reproducible signature is identified, subsequent studies could determine whether its magnitude or temporal evolution correlates with exposure severity or ocular findings before incorporating these data into multimodal predictive models. Until such a signal is established, this remains primarily a data-generation problem rather than a model-adaptation problem or hardware-deployment problem. Hardware optimization is a downstream consideration. The immediate requirement is establishing that relevant environmental or physiologic signals reliably predict ocular exposure or injury before determining how such a model should be implemented.

Management of chemical injury involves copious irrigation to normalize ocular surface pH to approximately 7.0. On the ISS, urine chemistry strips may be adapted for pH monitoring. Mild injuries are subsequently managed similarly to corneal abrasions with short-term topical anesthetics, topical antibiotics, lubricating drops, and cycloplegics. Topical tobramycin 0.3% with dexamethasone 0.1% may be used judiciously and tapered to control inflammation. Adjunctive oral vitamin C and doxycycline have also been recommended to promote collagen synthesis and reduce the risk of corneal ulceration and melting [30]. Given the urgency of irrigation after chemical exposure, the more plausible role for AI is upstream of treatment: integrating environmental and physiologic data to identify hazardous exposures and support early recognition, while established protocols continue to guide management once an injury is suspected.

### 3.4. Lens Dislocation

Whereas chemical injuries primarily affect the ocular surface, blunt mechanical trauma may disrupt deeper intraocular structures, including the crystalline lens. Lens subluxation is defined as partial displacement of the lens from its normal position due to incomplete disruption of the zonular fibers, leaving the lens decentered but partially supported and retained within the pupillary area. In contrast, lens dislocation refers to complete displacement of the lens from its normal position, with total loss of zonular support, so that the lens is entirely displaced into either the anterior chamber or the vitreous cavity. In penetrating and perforating eye injuries, the zonular fibers can break through a direct or indirect mechanism, whereas in blunt trauma, the zonular fibers are stretched or ruptured due to an equatorial expansion secondary to anterior–posterior compression of the globe [46]. Without timely intervention, severe complications, including progressive myopia, angle closure glaucoma, retinal tear, and vision loss, may occur [10,47].

Although no cases of spaceflight-related lens dislocation have been reported, blunt trauma aboard spacecraft can occur due to confined environments and floating objects. Crew members may collide with equipment or unsecured objects, including loose cables, cords, and hand-held tools, and sustain impact injuries. With sufficient mass and speed, the resulting damage from these impacts may lead to lens subluxation or displacement [48]. Besides mechanical causes, spaceflight-associated structural changes in the eye may also influence lens stability. In particular, several studies have documented reductions in anterior chamber depth and axial length following long-duration missions [49]. These findings are consistent with a mild anterior displacement or thickening of the lens. Reduced axial length decreases the focal distance, which shifts refraction toward hyperopia and may alter the posterior tension on the zonules that stabilize the lens [50]. These physiologic changes are also important diagnostically because the anatomy used to identify abnormal lens position on Earth may itself change during spaceflight.

Lens subluxation or displacement is typically diagnosed using slit-lamp examination after full pupillary dilation or with ocular ultrasound [51,52,53]. Although ultrasound imaging is available aboard spacecraft, accurate interpretation is constrained by the absence of trained ophthalmic specialists. Recent advances in artificial intelligence offer a compelling solution to this limitation. Cheng et al. developed a deep learning model based on ultrasound biomicroscopy images that reliably differentiated acute angle-closure eyes with and without lens subluxation, achieving diagnostic performance comparable to that of ophthalmologists. The CNN model delivered a strong overall performance, yielding a high AUROC of 0.9046 with the fastest image-processing time, highlighting its suitability for time-sensitive, resource-limited environments such as spaceflight [54]. This provides relatively direct evidence that CNNs can recognize imaging features associated with lens subluxation, although the model was developed in terrestrial patients with acute angle closure rather than traumatic lens displacement. Its rapid processing is relevant to potential onboard use, but processing speed alone does not establish hardware feasibility because computational footprint, power requirements, and performance on flight-compatible hardware were not evaluated.

AI-based analysis may also be valuable in detecting secondary complications such as angle-closure glaucoma, where early changes can be subtle and evolve. Yang et al. developed an AI model capable of automatically quantifying key anterior chamber angle parameters from OCT images, including angle opening distance and trabecular-iris space area. Reduced values of these parameters are associated with progressive angle narrowing and increased risk of elevated IOP and angle-closure glaucoma. The model demonstrated strong agreement with expert graders, supporting its reliability for clinical assessment [55]. The two models address complementary diagnostic tasks, as ultrasound-based CNN analysis provides direct evidence for identifying lens subluxation, while automated OCT may be more relevant to monitoring secondary angle narrowing. Both models require prospective validation for these purposes during spaceflight.

Physiologic domain shift represents a key limitation in translating the lens-subluxation model to spaceflight. The model by Cheng et al. learned what a normal lens position looks like from terrestrial patients, whereas spaceflight itself is associated with reduced anterior chamber depth and axial length. Applied without adjustment, the model could potentially misclassify spaceflight-associated anatomic change as lens subluxation or miss true displacement against an altered reference. One approach would be to establish an individualized preflight ultrasound biomicroscopy baseline for each astronaut and evaluate subsequent imaging relative to that baseline, similar to the use of preflight and postflight OCT measurements for other ocular changes. This would shift the diagnostic task from determining whether an eye differs from a terrestrial population toward detecting meaningful change from that individual’s preflight anatomy. The magnitude of expected in-flight variation would still need to be characterized so that physiologic adaptation can be distinguished from traumatic displacement.

If lens displacement is minimal and vision remains largely unaffected, management may consist of observation. In cases of associated complications such as elevated IOP, medical therapy may include IOP-lowering agents such as beta blockers, carbonic anhydrase inhibitors, alpha agonists, and prostaglandin analogs, although data on their effectiveness in the spaceflight environment remain limited [24]. AI may serve two distinct functions within this workflow by identifying structural changes suspicious for lens displacement after trauma and monitoring for secondary changes, such as progressive angle narrowing, that may warrant treatment or escalation.

### 3.5. Retinal Tears and Detachment

Trauma severe enough to destabilize the crystalline lens may simultaneously transmit force to the posterior segment, placing the retina at risk for vision-threatening injury. Retinal tears are full-thickness breaks in the neurosensory retina, promoting leakage of fluid from the vitreous cavity through the retinal breaks into the subretinal space, ultimately separating the retina from the retinal pigment epithelium (retinal detachment) [56]. While the most common cause of retinal tears is posterior vitreous detachment (PVD), which is itself age-dependent and occurs earlier in patients with myopia or after cataract surgery, other factors, including intraocular inflammation and trauma, are also known to predispose to retinal tears [57]. Symptoms such as new-onset floaters, flashes of light, and peripheral or central visual loss are often associated with PVD and may signal the presence of a retinal tear [56].

On Earth, sources of traumatic retinal detachment are numerous and may include occupational injuries, motor vehicle accidents, sports-related injuries, explosion-related injuries, head traumas, falls, and assaults [58,59,60]. While the sources of trauma may be different in space, astronauts are not exempt from these types of injuries. They, too, are at risk for ocular trauma due to unique environmental hazards and operational factors. These include accidental contact with equipment, floating debris, and unintentional impacts in microgravity, where objects move unpredictably, and even minor collisions can transmit force to the eye. Together, these hazards may cause closed or open-globe injuries, each leading to retinal detachment in their own distinct way [60].

Indirect ophthalmoscopy remains the gold standard for the detection and diagnosis of retinal tears and detachments [61], although ultra-widefield (UWF) imaging can be developed as a portable device to serve as a useful adjunct in diagnostic retinal imaging [62,63]. However, there remains a significant diagnostic gap due to the limitations of image interpretation by crew members. CNN-based analysis of UWF fundus imaging could address this gap by detecting structural abnormalities that predispose to retinal tears, including vitreoretinal traction, retinal thinning, and small retinal holes. More specifically, they can potentially be developed to detect retinal detachment severity along a graded scale from 0 to 5, ranging from no detachment (0), small localized peripheral detachment (1), larger peripheral detachment (2), extensive macula-on detachment (3), macula-off detachment (4), and total detachment with proliferative vitreoretinopathy or other complex features (5). Unlike several applications discussed above, retinal tears and detachments represent a predominantly image-based diagnostic task for which CNNs are well matched to the available terrestrial evidence. Clinically useful outputs extend beyond identifying abnormalities and include determining whether a retinal break is associated with detachment and whether the macula is involved, both of which influence urgency and prognosis.

Studies have already shown that CNNs built to detect and classify retinal breaks and detachments on UWF imaging have high accuracy with AUROCs of 0.913 for the detection and classification of retinal breaks and 0.972 for the detection and classification of retinal detachments, respectively. Discrimination between breaks without detachment, breaks with detachment, and detachments without breaks is also possible with these models. Separate models that distinguish between macula-on and macula-off detachments have also been developed with areas under the curve (AUCs) as high as 0.975 with a sensitivity of 93.8% and a specificity of 90.9% [62,64]. Among the ocular injuries reviewed, this represents relatively direct evidence because the models evaluate the same retinal pathology that would need to be recognized during spaceflight. However, these performance estimates were obtained using terrestrial UWF imaging and do not establish equivalent performance with a portable flight-compatible system or under spaceflight acquisition conditions. The existing evidence supports CNN-based image classification more directly than a multimodal approach for this task, although additional clinical inputs could contribute to triage.

Hardware feasibility presents a more immediate translational barrier for this application because both image acquisition and local inference must be adapted for onboard use. Terrestrial UWF cameras and the computers used to run these models are large, whereas a spaceflight system would need to be substantially smaller and operate with more limited onboard computing resources. Model quantization or pruning could reduce computational requirements, followed by testing to determine how much diagnostic performance is lost after compression. Because detection of a retinal detachment may trigger emergency evacuation, evaluation of compressed models should emphasize whether sensitivity is preserved for clinically consequential findings, particularly macula-on detachments in which delayed recognition may allow progression, rather than relying on overall accuracy alone. Image acquisition presents a separate source of domain shift. Without the chin rests and head-stabilization systems used in terrestrial clinics, motion artifact may degrade UWF images in ways the original models were not trained to interpret. Retraining or testing on deliberately motion-degraded images could help determine whether performance is maintained under more representative acquisition conditions. A flight-ready system would need to acquire sufficient-quality retinal images using portable equipment while also performing local CNN inference without clinically important loss of sensitivity.

Prompt intervention with laser photocoagulation, pneumatic retinopexy, scleral buckle, or pars plana vitrectomy is required to preserve vision. This ocular emergency should prompt emergency evacuation back to Earth given the lack of equipment and ophthalmologic training on the ISS. In this setting, the principal role of AI would be autonomous detection and triage rather than definitive management, helping distinguish findings that warrant observation or repeat imaging from a retinal tear or detachment requiring urgent Earth-based consultation and, when indicated, evacuation. Given the consequences of a missed detachment, validation for this use should place particular emphasis on minimizing false-negative assessments of vision-threatening disease.

### 3.6. Orbital Fractures

Not all vision-threatening injuries arise within the globe itself, as trauma involving the orbital skeleton presents an additional diagnostic challenge in resource-limited environments. Microgravity disrupts skeletal homeostasis by accelerating osteoclast-mediated bone resorption and suppressing osteoblast function. Increased expression of genes involved in osteoclast maturation and activity, along with elevated levels of collagen degradation products, reflects enhanced bone breakdown, while impaired osteoblast cytoskeletal integrity further inhibits new bone formation [65,66]. This imbalance contributes to a heightened fracture risk in astronauts, including orbital fractures. Although rare, this form of ocular injury may occur following blunt impact. Of note, there is one reported incident in which an astronaut sustained a left infraorbital contusion after striking the galley oven door aboard a spacecraft [67].

On Earth, non-contrast computed tomography (CT) of the face and orbit is the gold standard for detecting orbital fractures [68]. However, CT involves ionizing radiation, which is particularly concerning for radiosensitive structures such as the lens and increases the risk of radiation-induced cataract formation [69,70]. Ultrasonography provides a radiation-free alternative and has demonstrated promising diagnostic performance. In one study, ultrasound detected 34 of 39 orbital fractures and correctly identified all 81 intact orbits, while another reported 86% accuracy and 85% sensitivity for orbital floor fractures [71,72]. Support for ultrasound-based fracture detection is further strengthened by evidence from non-ocular skeletal trauma. Studies of pediatric forearm fractures have shown ultrasound performing comparably to X-ray while offering advantages in speed, comfort, and cost [73]. A meta-analysis of distal forearm fractures reported pooled sensitivity and specificity of 97% and 95%, respectively, and noted that fractures detected on ultrasound but missed on x-ray were often misclassified as false positives, suggesting that ultrasound accuracy may be underestimated across fracture types [74]. Although ultrasound remains less sensitive than CT for orbital trauma, these findings support its potential when definitive imaging is unavailable and provide a basis for investigating whether automated interpretation could improve its utility during spaceflight.

Building on these advances, AI-enhanced ultrasound systems are promising in facilitating fracture detection in resource-limited or imaging-restricted environments such as spaceflight, where operator experience and image quality may be highly variable. While current systems have not been applied directly to orbital fractures, their foundational techniques address core limitations of ultrasound interpretation, including artifact suppression, operator dependence, and difficulty identifying subtle cortical discontinuities. Tripathi et al. developed an unsupervised deep-learning framework that applies adaptive time-gain attenuation to suppress superficial brightness artifacts and enhance visualization of deeper osseous interfaces, followed by the generation of bone probability maps that highlight regions of structural discontinuity. The model further identifies key points, which are landmarks corresponding to abrupt changes in bone morphology, enabling automated localization of suspected fracture sites without reliance on predefined fracture labels [75]. In parallel, CNN models trained on large ultrasound datasets have demonstrated reliable detection of wrist and elbow fractures, achieving accuracies of 0.889 and 0.750, respectively, supporting the feasibility of learning fracture-relevant echogenic patterns across skeletal regions [76]. Existing AI models for ultrasound fracture detection are trained to recognize fundamental signs of osseous injury, including disrupted cortical contours and altered bone-soft tissue acoustic interfaces. Because these markers appear across different bones, similar models could potentially be adapted for orbital applications with appropriate training data. The available models also address different aspects of the diagnostic task. The framework developed by Tripathi et al. focuses on image enhancement and localization of structural discontinuities, while fracture-classification CNNs identify patterns associated with osseous injury. These functions could potentially complement one another, but neither has demonstrated reliable detection of orbital fractures. Since CT imaging is unlikely to be routinely available aboard future exploration-class spacecraft, AI-enhanced ultrasonography may offer a potential approach for evaluating orbital trauma during long-duration missions.

The immediate question is whether these approaches transfer from limb fractures to the substantially different anatomy of the orbit. One approach would be to retrain or fine-tune an existing model using orbital fracture ultrasound images from emergency departments or facial trauma clinics and validate it in an independent orbital trauma cohort. Performance should be evaluated not only for overall fracture detection but also for clinically important findings such as fracture location and features associated with extraocular muscle entrapment. If adequate orbital performance is demonstrated, subsequent testing could determine whether accuracy is preserved after model compression for onboard computation and when images are acquired under less controlled conditions, including variable probe positioning and operator experience. This sequence would establish orbital validity before introducing the additional computational and image-acquisition constraints of spaceflight.

Orbital swelling may be managed with NSAIDs and acetaminophen. Corticosteroids may be used for additional inflammation control. Although not all orbital fractures require surgical intervention, surgical repair, when indicated, should ideally be performed within two weeks. Urgent evacuation should be considered in cases of enophthalmos, persistent diplopia, ocular motility restriction, or suspected extraocular muscle entrapment, which may also trigger the oculocardiac reflex. AI-assisted ultrasound could support these decisions when CT and specialist interpretation are unavailable by helping identify suspected orbital injury and findings that warrant urgent evacuation.

### 3.7. Barotrauma

Beyond direct blunt trauma, alterations in environmental pressure represent another mechanism capable of producing orbital and optic nerve injury during spaceflight. Barotraumas refer to damage to the body’s tissues caused by the pressure differences between internal air cavities (such as sinus cavities, ears, lungs, and digestive tract) and the surrounding environment when the body moves to or from higher pressures. In space, these events can arise from explosions, failed environmental control systems, cabin breaches, leaks, or the rapid ejection of crew from a pressurized environment. When ambient pressure suddenly drops or rises, it causes gas filled spaces in the body to expand and compress, resulting in damage via shear stress or stretching. Although systemic injuries (pulmonary, auditory, or neurologic) often accompany pressure changes, the eye and orbit are uniquely vulnerable because they contain adjacent tissues filled with air, rigid bony boundaries, and delicate neurovascular structures [77].

Although spaceflight specific data are limited, analogous injuries are well described in diving contexts and offer important insight into potential ophthalmic complications such as periorbital ecchymosis, eyelid and orbital edema, subconjunctival hemorrhage, hyphema, and orbital hematoma [78,79]. Other than dive-related cases, a notable example involves a U.S. Air Force aviator who experienced in-flight ocular pain, redness, and proptosis, later found to have an old medial orbital wall fracture presumably secondary to expanding ethmoid sinus gas at high altitude. The event resulted in optic nerve pallor, cupping, and a persistent visual defect, consistent with barotrauma-induced optic neuropathy [80]. The evidence for ocular barotrauma during spaceflight is therefore indirect, with potential injury patterns inferred primarily from diving and aviation rather than documented spaceflight cases.

Ocular barotrauma in space represents a critical intersection of neuro-ophthalmic risk and environmental pressure management. Some consequences, such as subconjunctival or orbital hemorrhage, periorbital swelling, exophthalmos, and transient diplopia, may resolve with conservative management [10]; however, more severe sequelae involving the optic nerve can lead to persistent visual deficits, highlighting the need for early detection and longitudinal monitoring. Unlike retinal detachment or lens subluxation, however, the literature cited here does not provide a dedicated AI model for detecting ocular barotrauma or barotrauma-induced optic neuropathy. The potential role of AI must consequently be inferred from its ability to analyze the structural changes that barotrauma may produce rather than from direct evidence in this condition.

Compact OCT systems deployed onboard spacecraft could play a central role in this effort by providing cross-sectional imaging of the optic nerve head, retinal nerve fiber layer, ganglion cell complex, and peripapillary retina. These structures are particularly vulnerable to pressure-related injury, edema, disruptions to optic nerve signal transport, and ischemic compromise. CNN models can be trained to analyze serial OCT scans for subtle structural changes that may be difficult to detect by non-specialist crew members, including early disc edema, retinal nerve fiber layer thickening or thinning, optic nerve head contour changes, and progressive ganglion cell loss. Such systems could generate automated risk scores, compare current scans with prior baseline images, and flag concerning trends suggestive of evolving optic neuropathy. For this application, longitudinal change may be more informative than classification from a single image because the diagnostic task is detecting new structural injury after a pressure-related event. Individual preflight OCT measurements could provide a reference for serial comparison after suspected exposure. This could shift optic nerve evaluation from episodic examinations toward continuous structural surveillance, allowing earlier detection of pressure-related changes before irreversible visual loss occurs. A multimodal approach may eventually offer additional value by combining OCT changes with the magnitude and duration of the pressure event, visual symptoms, and other clinical findings, although the reviewed evidence does not establish the performance of such a system.

Spaceflight physiology complicates this approach because optic nerve and retinal structural changes can occur during long-duration missions independent of trauma. An algorithm trained on terrestrial OCT could potentially attribute these background changes to acute pressure-related injury. Before clinical use, a model would need to distinguish expected in-flight variation from changes temporally associated with a barometric event, ideally using longitudinal astronaut data and individualized baselines. This limitation is especially important here because no barotrauma-specific ocular AI model currently provides a starting point for direct adaptation. The evidence is consequently less mature than for retinal detachment or corneal abrasion, and the immediate research need is to define the imaging phenotype of pressure-related ocular injury before optimizing a model for onboard deployment.

Barotrauma in spaceflight is primarily prevented through carefully controlled pressure transitions, since rapid changes in cabin or suit pressure can create harmful gradients across the middle ear and sinuses. NASA protocols emphasize slowing these transitions and allowing them to be paused or reversed if a crewmember develops symptoms. If barotrauma is severe, management may require escalation through ground-based flight surgeon consultation. AI-assisted ocular imaging could supplement this assessment by identifying structural changes that warrant closer monitoring or specialist review, but current evidence does not support autonomous diagnosis or management of ocular barotrauma. The diagnostic challenges, supporting AI approaches, and potential spaceflight applications in ocular trauma reviewed above are summarized (Table 1).

## 4. Challenges to AI Applications in Space Medicine

The integration of AI into medical diagnostics for spaceflight is constrained by the limited volume and narrow diversity of available space-relevant biomedical data. Only a small subset of humans has ever traveled beyond Earth, and an even smaller subset has undergone high-quality ocular imaging suitable for training robust AI models. Such data scarcity limits model generalizability and increases the risk of bias. New initiatives to build open biomedical data repositories for commercial spaceflight, combined with the anticipated growth of the space tourism sector, may help address this gap by increasing the number of spaceflight participants and expanding the pool of health data available for algorithm development. As these datasets mature, they may enable AI systems that better reflect the physiological variabilities of spacefarers [81]. For the ocular-trauma applications considered in this review, the limitation is even greater because relatively uncommon traumatic events must be captured within an already small spaceflight population. Terrestrial datasets will likely remain necessary for initial model development, while spaceflight data may be most valuable for adaptation and validation.

On a similar note, another major challenge involves the quality and applicability of data used to train AI systems meant for use in space medical care. Current algorithms rely predominantly on ground-based datasets that reflect physiology and environmental conditions on Earth, which may not hold true in microgravity. Among many changes, spaceflight induces shifts in cardiovascular regulation, immune function, fluid distribution, and neuro-ocular physiology that may alter disease presentation and change the diagnostic features AI systems rely upon on Earth. Archived spaceflight medical data could supplement training, but these records often lack consistency because crew members frequently underreport symptoms or defer scheduled health monitoring in order to remain flight-eligible or avoid operational delays. The push for open spaceflight datasets may also help address this concern [81]. This domain shift has different implications across the applications reviewed above. Corneal models may encounter unfamiliar illumination, dust, and image quality, lens and optic nerve assessment may be confounded by spaceflight-associated structural changes, and models adapted from non-ocular injuries must first establish validity in the relevant ocular anatomy. Addressing domain shift will consequently require application-specific validation rather than a single correction applicable across all ocular-trauma models.

Ethical, regulatory, and privacy constraints also present substantial barriers. Because space crews are small and easily identifiable, medical information collected during missions is difficult to fully anonymize, complicating its use for training or refining AI systems. As with any emerging medical technology, the deployment of AI in space requires clear, voluntary, and well-informed consent, particularly since crew members will effectively serve as first-in-human users of these systems in an isolated environment. They must be thoroughly briefed on the uncertainties, experimental nature, and potential risks associated with AI-supported care. Continuous algorithmic updating adds another layer of complexity. As spacecraft increasingly incorporate autonomous diagnostic tools, these systems need updated data inputs to remain accurate and relevant. Such ongoing data collection again hinges on astronaut willingness to share sensitive health information. Without explicit consent from crew members, the maturation of space-relevant AI systems becomes challenging. Additionally, if an AI-generated recommendation contributes to medical harm, questions persist about accountability and how responsibility should be distributed between crew, mission control, system designers, and the AI itself, issues that remain unresolved in current legal frameworks [82]. These concerns become particularly consequential as AI moves from identifying an abnormal image toward recommending observation, treatment, or emergency escalation. Greater clinical autonomy requires correspondingly stronger validation, predefined limits on model authority, and mechanisms for communicating uncertainty to crew members.

Beyond the data-scarcity and ethical challenges, translating any model into an onboard spaceflight tool faces computational footprint and imaging quality challenges. Spacecraft computing hardware operates under severe size, weight, and power limitations, while many models discussed in this review were developed using terrestrial clinical-grade computing. Model compression by quantization or pruning may reduce computational requirements, but compressed models would need to be retested to determine whether clinically important performance is preserved, particularly sensitivity for injuries in which missed detection could threaten vision. Imaging acquired during spaceflight may also differ systematically from terrestrial training data because of motion artifact, smaller or lower-fidelity portable sensors, non-standardized illumination, and variable positioning. Existing terrestrial images could be deliberately modified with simulated motion blur, sensor noise, and altered illumination before retraining to test model robustness under more representative conditions. Importantly, hardware feasibility and diagnostic performance cannot be considered independently. A model that performs well using clinical-grade imaging and computing but loses sensitivity after deployment on portable hardware would not be suitable for autonomous use. Validation should ultimately reproduce the complete intended workflow, including the onboard imaging device, compressed model, acquisition conditions, and decisions expected of the crew. The current evidence maturity, principal translational barriers, and priority next steps for AI-assisted ocular trauma care are summarized (Table 2).

## 5. Conclusions

AI may expand the ability of astronauts to recognize and triage ocular trauma when specialist expertise and timely Earth-based support are unavailable, but translational readiness varies substantially across applications. Models with relatively direct terrestrial validation exist for image-defined injuries such as retinal tear or detachment and lens subluxation, while other applications rely on less direct evidence or remain conceptual. Suspected globe penetration may benefit from multimodal assessment because no single finding reliably excludes injury, chemical injury requires establishment of relevant environmental or physiologic signals before predictive modeling is justified, and orbital fracture models require validation in orbital anatomy before spaceflight adaptation. Barotrauma remains earlier still, as the imaging phenotype of pressure-related ocular injury must first be better defined. Importantly, AI-based ocular diagnostic systems discussed in this review have not yet been validated during spaceflight, and all reported performance derives from terrestrial data. The current literature should therefore be viewed as defining a spaceflight validation agenda rather than demonstrating readiness for onboard clinical deployment.

Closing this gap will require development strategies matched to the maturity of each application. More established image-based models can progress toward testing with spaceflight-representative image degradation, portable imaging systems, model compression, and benchmarking on hardware representative of spacecraft constraints. Less mature applications should first establish the biological, imaging, or anatomic evidence necessary to justify model development. Ultimately, validation should evaluate the complete system that astronauts would use, including image acquisition, local computation, model performance, uncertainty communication, and the clinical decisions generated from its output. Privacy, informed consent, and accountability will also require clear frameworks as these systems assume greater autonomy. The goal is therefore not simply to reproduce terrestrial diagnostic accuracy in space, but to develop AI systems capable of extending ophthalmic decision-making into environments where specialist assessment may be delayed or unavailable.

## Figures and Tables

**Table 1:** AI Approaches and Potential Applications Across Ocular Injuries Relevant to Spaceflight. This table compares the diagnostic challenges associated with major ocular injuries relevant to spaceflight with the AI approaches best suited to each task, the supporting terrestrial evidence, and their potential roles during future missions. It highlights how CNN-based image analysis may support structural injury detection, while multimodal approaches may be more useful when imaging must be interpreted alongside clinical, physiologic, environmental, or exposure data.

Ocular Injury	Diagnostic Challenge During Spaceflight	Most Relevant AI Approach	Supporting Evidence	Potential Role During Spaceflight
**Corneal abrasion,** **Foreign body**	Epithelial defects and small foreign bodies may be difficult to identify without slit-lamp biomicroscopy. Portable imaging may also be affected by dust, variable illumination, and image quality.	CNN analysis of fluorescein-enhanced or anterior-segment imaging. Multimodal models could incorporate recent EVA or dust-exposure history.	CNNs detected and quantified epithelial defects from fluorescein-enhanced images with 91.2% accuracy. CorneAI improved anterior-segment diagnostic accuracy by 9.6%, including with smartphone images.	Detect and characterize epithelial injury, estimate severity, and support decisions regarding irrigation, treatment, repeat imaging, or escalation.
**Globe penetration**	Subtle wounds and preserved visual function may produce false reassurance. Ultrasound has limited sensitivity and must be performed cautiously.	Multimodal models integrating ocular imaging with visual acuity, wound characteristics, IOFB status, and other clinical findings.	EE-Explorer achieved an AUROC of 0.982 for vision-threatening ocular conditions. Other multimodal ocular-trauma models have predicted visual outcomes.	Generate graded risk estimates for suspected globe penetration and prioritize injuries requiring immediate stabilization and evacuation.
**Chemical injury**	Some hazardous exposures, particularly gaseous compounds, may be difficult to detect before clinically apparent injury develops.	Multimodal systems integrating environmental sensing, physiologic monitoring, and ocular findings.	Breath and VOC analysis can distinguish several terrestrial disease states. No evidence currently establishes a breath or physiologic signature specific to chemical ocular injury.	Potentially identify hazardous exposure patterns and prompt ocular assessment or preventive action before severe injury develops.
**Lens subluxation or dislocation**	Ultrasound is available onboard, but interpretation may require expertise. Normal spaceflight-related changes in ocular anatomy could complicate assessment.	CNN analysis of ultrasound biomicroscopy, supplemented by automated OCT assessment of secondary angle changes.	A CNN differentiated acute angle-closure eyes with and without lens subluxation with an AUROC of 0.9046. AI-based OCT analysis has reliably quantified anterior chamber angle parameters.	Detect lens displacement and monitor structural changes or secondary angle narrowing, ideally relative to an astronaut’s preflight baseline.
**Retinal tear or** **detachment**	Retinal tears and detachments require rapid recognition, while interpretation of peripheral retinal imaging may be difficult for non-specialist crew members.	CNN analysis of UWF retinal imaging.	UWF-based CNNs achieved AUROCs of 0.913 for retinal breaks and 0.972 for retinal detachments. Separate models distinguished macula-on from macula-off detachment with AUCs up to 0.975.	Detect retinal breaks or detachments, assess macular involvement, and support decisions regarding urgent consultation or evacuation.
**Orbital fracture**	CT may not be available during exploration-class missions. Ultrasound is a plausible alternative but remains operator-dependent and less sensitive than CT.	AI-enhanced ultrasound using feature extraction and CNN-based fracture detection.	AI ultrasound models can identify fracture-related cortical abnormalities, but current evidence is derived primarily from non-orbital skeletal injuries.	Assist non-specialists in identifying suspected orbital fractures and potentially recognizing injuries requiring evacuation.
**Barotrauma or** **pressure-related optic neuropathy**	Optic nerve injury may evolve after pressure changes and must be distinguished from other spaceflight-associated ocular changes.	Longitudinal OCT analysis, with future multimodal integration of imaging, symptoms, and pressure-exposure data.	No dedicated AI model has been validated for ocular barotrauma. OCT and AI provide a potential basis for serial structural monitoring.	Compare post-event imaging with individualized baselines and flag structural changes concerning for evolving optic neuropathy.

**Table 2:** Evidence Maturity, Translational Barriers, and Research Priorities for AI-Assisted Ocular Trauma Care in Spaceflight. This table compares the maturity of evidence across AI applications for ocular trauma and identifies the principal barrier, priority next step, and translational goal for each injury. It distinguishes applications with relatively mature terrestrial foundations that primarily require spaceflight-specific adaptation and operational validation from those that first require anatomic transfer, signal discovery, or definition of a reproducible imaging phenotype before meaningful AI development can proceed.

Ocular Injury	Evidence Maturity	Key Limitation to Translation	Priority Next Step	Translational Goal
**Corneal abrasion,** **Foreign body**	**Relatively mature**	Relevant terrestrial models exist, but performance under dust exposure, uneven illumination, sensor noise, and portable image acquisition is unknown.	Evaluate abrasion-specific models using spaceflight-representative image degradation and flight-compatible imaging systems. Test whether EVA or exposure history improves performance when incorporated as an additional input.	Preserve reliable epithelial-defect detection under realistic onboard imaging conditions.
**Globe penetration**	**Intermediate**	Terrestrial multimodal models provide relevant evidence, but injury mechanisms may differ during spaceflight and false-negative classification carries particularly high consequences.	Validate models on atypical trauma patterns and emphasize sensitivity, uncertainty calibration, and false-negative performance rather than overall accuracy alone.	Produce a graded risk assessment capable of identifying injuries that require immediate stabilization or evacuation.
**Chemical injury**	**Early/conceptual**	The biological and environmental signals needed for prediction have not been established. There is no evidence that ocular chemical injury produces a detectable breath signature.	First determine whether relevant chemical exposures produce reproducible environmental, physiologic, or ocular signals, potentially using terrestrial occupational-exposure cohorts.	Establish a valid signal for exposure or impending injury before developing predictive AI models.
**Lens subluxation or** **dislocation**	**Relatively mature**	A terrestrial model may misinterpret normal spaceflight-associated changes in anterior chamber depth or axial length as pathology.	Establish individualized preflight imaging baselines and evaluate whether models can distinguish traumatic change from expected spaceflight-related variation.	Detect clinically meaningful change from an astronaut’s own baseline rather than relying solely on terrestrial population norms.
**Retinal tear or** **detachment**	**Most mature among reviewed applications**	Strong pathology-specific terrestrial models exist, but UWF acquisition and model computation must be adapted to spacecraft constraints without sacrificing sensitivity.	Test portable retinal acquisition under motion and positioning limitations. Compress models through approaches such as quantization or pruning and determine whether clinically important detachments are missed after compression.	Translate established image-recognition capability into a reliable flight-compatible system for urgent retinal triage.
**Orbital fracture**	**Early/indirect**	Current AI evidence largely concerns fractures outside the orbit, so transferability to orbital anatomy remains unproven.	Adapt and externally validate fracture-detection models using orbital ultrasound datasets before addressing spaceflight-specific image degradation and hardware constraints.	Establish orbital validity first, followed by development of a portable autonomous ultrasound assessment system.
**Barotrauma or** **pressure-related optic neuropathy**	**Conceptual**	No dedicated ocular AI model exists, and the structural imaging phenotype of pressure-related injury must be distinguished from background spaceflight-associated ocular changes.	Characterize longitudinal OCT findings associated with pressure events and determine how they differ from expected in-flight variation using individualized baselines.	Define a reproducible imaging phenotype that can eventually support automated longitudinal surveillance.

## Data Availability

No new data were created or analyzed in this study. Data sharing is not applicable to this article.

## Abbreviations

The following abbreviations are used in this manuscript:

AI	Artificial Intelligence
AS-OCT	Anterior Segment Optical Coherence Tomography
AUC	Area Under the Curve
AUROC	Area Under the Receiver Operating Characteristic Curve
CNN	Convolutional Neural Network
CT	Computed Tomography
EVA	Extravehicular Activity
IOFB	Intraocular Foreign Body
IOP	Intraocular Pressure
ISS	International Space Station
LSAH	Lifetime Surveillance of Astronaut Health
LSDA	Life Sciences Data Archive
NASA	National Aeronautics and Space Administration
NSAID	Nonsteroidal Anti-Inflammatory Drug
OCT	Optical Coherence Tomography
PVD	Posterior Vitreous Detachment
SHT	Space Shuttle
UWF	Ultra-Widefield
VOC	Volatile Organic Compound
